# Virus infection drives IL-2 antibody complexes into pro-inflammatory agonists in mice

**DOI:** 10.1038/srep37603

**Published:** 2016-11-25

**Authors:** Wendy W. L. Lee, Teck-Hui Teo, Fok-Moon Lum, Anand K. Andiappan, Siti Naqiah Amrun, Laurent Rénia, Olaf Rötzschke, Lisa F. P. Ng

**Affiliations:** 1 Singapore Immunology Network, Agency for Science, Technology and Research, Singapore (A*STAR), Singapore 138648, Singapore; 2 NUS Graduate School for Integrative Sciences and Engineering, National University of Singapore, Singapore 117456, Singapore; 3 Department of Biochemistry, Yong Loo Lin School of Medicine, National University of Singapore, Singapore 117597, Singapore; 4 Department of Microbiology, Yong Loo Lin School of Medicine, National University of Singapore, Singapore 117597, Singapore

## Abstract

The use of IL-2/JES6-1 Ab complex (IL-2 Ab Cx) has been considered as a potential therapeutic for inflammatory diseases due to its selective expansion of regulatory T cells (Tregs) in mice. Here, IL-2 Ab Cx was explored as a therapeutic agent to reduce joint inflammation induced by chikungunya virus, an alphavirus causing debilitating joint disease globally. Virus-infected mice treated with IL-2 Ab Cx exhibited exacerbated joint inflammation due to infiltration of highly activated CD4^+^ effector T cells (Teffs). Virus infection led to upregulation of CD25 on the Teffs, rendering them sensitive towards IL2 Ab Cx. Ready responsiveness of Teffs to IL-2 was further demonstrated in healthy human donors, suggesting that the use of IL-2 Ab Cx in humans is not suitable. Changes in IL-2 sensitivity during active virus infection could change the responsive pattern towards the IL-2 Ab Cx, resulting in the expansion of pro-inflammatory rather than anti-inflammatory responses.

Recruitment and activation of the immune system in response to virus infections result in the release of potent cytokines and cytotoxic molecules^1^,^2^,^3^. While these molecules are important in clearing the virus, they can also cause tissue damage when released at inappropriate levels, as shown in influenza virus and chikungunya virus infections^1^,^2^,^3^,^4^,^5^,^6^,^7^,^8^.

CHIKV is an arthropod-borne alphavirus that re-emerged in late 2005 and caused explosive outbreaks of chikungunya fever (CHIKF) around the world^9^,^10^,^11^,^12^. Infected patients experienced fever and extremely painful incapacitating polyarthralgia that may persist for months^13^. There are no commercially available vaccines and antivirals. Due to the lack of targeted treatments, patients are only given palliative care to relieve the symptoms^14^. Studies have provided evidence that the virus-induced joint inflammation is immune-mediated^5^,^15^,^16^,^17^. Thus, immunotherapy could provide a targeted treatment for this inflammatory disease. One mechanism used by the immune system to restrain excessive pro-inflammatory control is the induction of regulatory T cells (Tregs), a subset of CD4^+^ T cells that limit tissue damage induced during virus infection^18^,^19^,^20^,^21^.

In mice, Tregs can be expanded by application of the preformed IL-2/antibody complex, IL-2/JES6-1 (IL-2 Ab Cx). The injection of IL-2 Ab Cx results in a drastic increase in the number of circulating Tregs^22^. Prophylactic treatment using IL-2 Ab Cx has been shown to be effective in alleviating various immunopathologies including herpetic stromal keratitis, malaria and experimental autoimmune encephalomyelitis^19^,^23^,^24^. In our previous study using a CHIKV infection mouse model, prophylactic treatment with IL-2 Ab Cx also abrogated CHIKV-induced joint inflammation through selective expansion of Tregs^21^,^22^. While this intervention was highly effective, IL-2 Ab Cx was injected prophylactically prior to virus infection. In a more relevant setting, we therefore explored the potential of IL-2 Ab Cx as a therapeutic agent in this study. Interestingly, despite the increased number of circulating Tregs upon IL-2 Ab Cx administration after manifestation of inflamed joints, the treatment was counter-productive. Animals suffered from an aggravated inflamed joint pathology associated with an increased infiltration of activated CD4^+^ effector T cells (Teffs). Profiling of Teffs in the draining lymph node indicated up-regulation of CD25, thus rendering them sensitive towards IL-2 Ab Cx. Administration of IL-2 Ab Cx at this point therefore led to a massive expansion of virus-specific Teffs, which then migrate to the infected joint footpad and aggravate inflamed joint pathology. We show that active virus infection can change the response pattern of IL-2 Ab Cx, resulting in the expansion of pro-inflammatory rather than anti-inflammatory T cells, cautioning its use as a therapeutic agent.

## Results

### Administration of IL-2 Ab Cx after virus infection exacerbates inflamed joint pathology

It was previously shown that prophylactic IL-2 Ab Cx treatment effectively mediates the expansion of Tregs, resulting in the alleviation of CHIKV-induced inflamed joints^21^. However, for this therapy to be clinically relevant, administration of IL-2 Ab Cx also has to be effective when given after the manifestation of clinical symptoms^4^,^25^,^26^,^27^. The incubation period for CHIKV typically ranges from 3–14 days in patients and it is during this period where patients normally seek treatment to alleviate the symptoms^28^. Therefore, therapeutic administration of IL-2 Ab Cx was chosen on 3–5dpi to represent the clinical setting.

**Figure 1:**
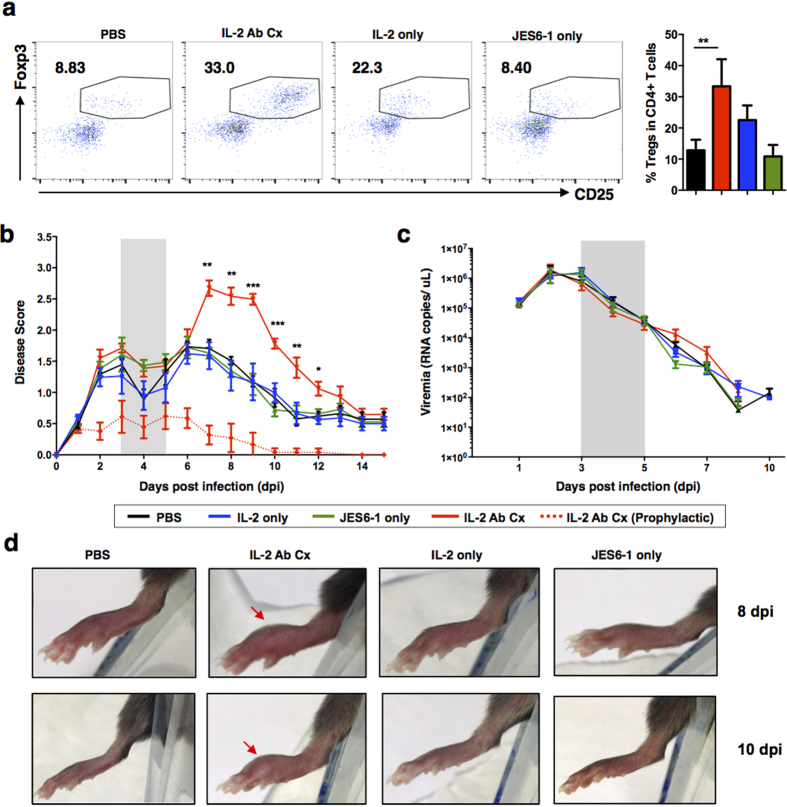
Therapeutic treatment with IL-2 Ab Cx exacerbates inflamed joint pathology. WT mice (n = 5 per group) were infected s.c with 10^6^ PFU CHIKV. PBS, IL-2 Ab Cx, IL-2 only or JES6-1 only was administered to CHIKV-infected mice on 3, 4 and 5 dpi. **(a)** Scatterplots show CD4^+^ T cells expression of Foxp3 versus CD25. Population in the boxed region denotes Foxp3^+^CD25^+^ Tregs with the indicated percentage Tregs in total CD4^+^ T cells. Bar chart on the right shows average percentage of Tregs in total CD4^+^ T cells in the blood. **(b)** Joint swelling was monitored daily from 1–15 dpi. Mice treated prophylactically with IL-2 Ab Cx were included as reference. **(c)** Viremia was determined from blood collected from the tail vein from 1–10 dpi. Areas shaded in grey indicate the time-points (3, 4 and 5 dpi) where respective treatments were administered. **(d)** Representative images show joint footpad swelling at 8 and 10 dpi in CHIKV-infected mice. Arrows indicate area of persistent swelling. Statistical analysis was done across all CHIKV-infected groups using Kruskal Wallis test, followed by Dunn post-test comparing to PBS. All data are presented as mean ± SD and from 2 independent experiments. (**p = 0.0018, 7 dpi joint swelling IL-2 Ab Cx, **p = 0.0010, 8 dpi joint swelling IL-2 Ab Cx, ***p = 0.0007, 9 dpi joint swelling IL-2 Ab Cx, ***p = 0.0009, 10 dpi joint swelling IL-2 Ab Cx, **p = 0.0028, 11 dpi joint swelling IL-2 Ab Cx, **p = 0.0055, 12 dpi joint swelling IL-2 Ab Cx).

Although the administration of IL-2 Ab Cx increased the levels of circulating Tregs (Fig. 1a and Fig. S1), this did not translate to the protective response that was observed in the prophylactic setting^21^. Instead, an exacerbation of the inflamed joint was observed (Fig. 1b). While the administration of IL-2 Ab Cx did not affect the course of viremia (Fig. 1c), the exacerbation in pathology took place between 7 to 10 dpi when the control groups (PBS, IL-2 only, JES6-1 only) were already starting to recover (Fig. 1b,d). This observation is in sharp contrast to the striking reduction of virus-induced joint footpad swelling observed during prophylactic treatment (Fig. 1d)^21^.

### Therapeutic treatment with IL-2 Ab Cx causes infiltration of CD25-expressing CD4^+^Foxp3-Teffs

**Figure 2:**
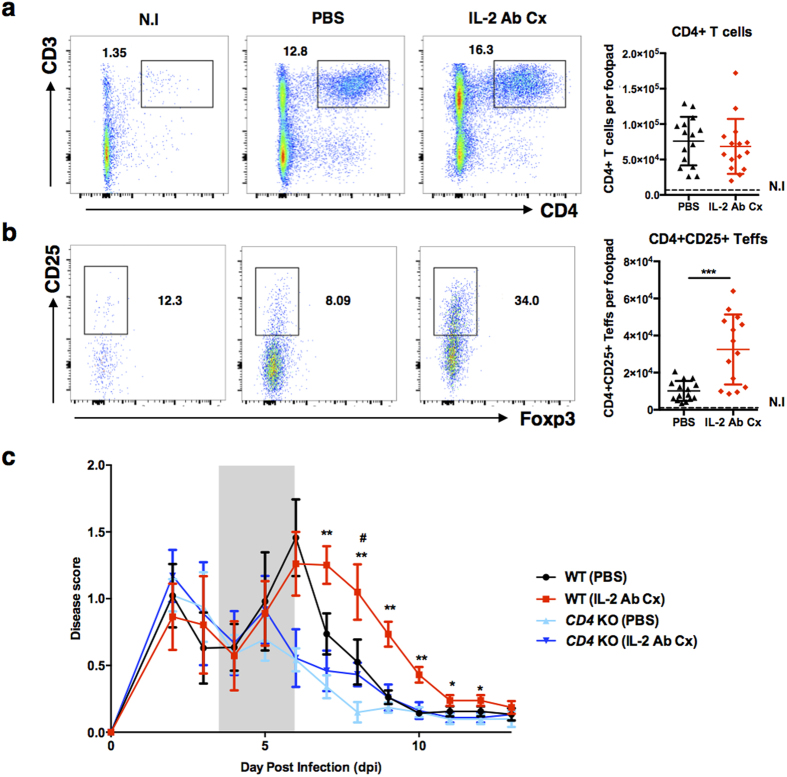
Exacerbated inflamed joint pathology in therapeutically treated mice is caused by increased infiltration of CD4^+^CD25^+^ Teff cells. WT mice (n = 5 per group) were infected s.c with 10^6^ PFU CHIKV and administered with either PBS or IL-2 Ab Cx. Joint footpad cells from treated animals were isolated on 7 dpi and analyzed by flow cytometry. Dead cells were excluded using Live/dead staining. (**a**) Representative scatterplots show CD3 and CD4 expression gated on CD45^+^ leukocytes. Numbers in scatter plots indicate percentage of CD3^+^CD4^+^ population in total CD45^+^ cells. Scatter dot plot (right) shows average number of CD3^+^CD4^+^ T cell infiltrates per infected joint footpad. (**b**) Representative scatterplots show CD25 and Foxp3 expression gated on CD3^+^CD4^+^ cells from (**a**). Numbers in scatterplots indicate the percentage of CD25^+^ cells in total CD3^+^CD4^+^ T cells from (**a**). Scatter dot plot shows average number of CD25+ Teffs per infected joint footpad. All data are presented as mean ± SD and from 3 independent experiments. Statistical analysis was performed using two-tailed Mann Whitney *U* test comparing between PBS and IL-2 Ab Cx. (***p = 0.0006 CD4^+^CD25^+^ Teffs). (**c)** IL-2 Ab Cx or PBS (n = 5 per group) was administered to CHIKV-infected wildtype (WT) or *CD4* knockout (KO) mice on 3, 4 and 5 dpi. Joint swelling was monitored daily from 1–13 dpi. All data are presented as mean ± SD and representative of 2 independent experiments. Statistical analysis was performed using two-tailed Mann Whitney *U* test comparing between WT (PBS) and WT (IL-2 Ab Cx), or, *CD4* KO (PBS) and *CD4* KO (IL-2 Ab Cx). (**p = 0.0079 WT 7dpi, **p = 0.0079 WT 8 dpi, **p = 0.0079 WT 9 dpi, **p = 0.0079 WT 10 dpi, *p = 0.0079 WT 8 dpi, *p = 0.0238 WT 9 dpi, *p = 0.0238 WT 10 dpi, *#p* = 0.0286 *CD4* KO 8 dpi).

Experiments in *CD4*-knockout (KO) mice indicated that CD4^+^ Teffs are important drivers of CHIKV-induced pathology^4^. We therefore analyzed the CD4^+^ T cells infiltrating the footpad. Cells were profiled on 7 dpi when differences in the inflamed joint pathology between treated and non-treated mice were most striking (Fig. 1b). Although there was no significant difference in the number of infiltrating CD4^+^ T cells in the joint footpad of IL-2 Ab Cx-treated mice (Fig. 2A), a significant increase in the number of infiltrating CD4^+^Foxp3^−^ T effector cells (Teffs) expressing CD25 was noted (Fig. 2b).

CD25 is the α-chain of the IL-2 receptor and confer high affinity of the receptor complex for IL-2. While it is the key-marker of Treg cells, CD25 is also expressed by conventional CD4^+^ Teffs upon activation^29^,^30^. Indeed, cytometry analysis of popliteal lymph nodes (pLNs) from virus-infected mice showed a significant increase in the expression of CD25 on Teffs (Fig. S2a and b). Notably, expression of CD122 (IL-2Rβ) was also regulated on these Teffs (Fig. S2c). Upregulation of these IL-2 receptor chains is likely to render the Teffs more sensitive to the presence of IL-2 Ab Cx.

The significant increase in CD25^+^ Teffs in the footpad of CHIKV-infected mice suggests that exposure to IL-2 Ab Cx during virus infection promote their expansion and resulted in exacerbated joint inflammation (Fig. 1b). In contrast, other subsets that were examined showed no significant difference between the control and IL-2 Ab Cx-treated groups (Fig. S3). To confirm the involvement of CD4^+^ T cells, experiments were repeated in *CD4* KO mice. As expected, there was no difference in the inflamed joint pathology between non-treated and IL-2 Ab Cx-treated *CD4* KO mice (Fig. 2c). This strongly suggests that it is the infiltrating CD4^+^CD25^+^ Teffs that were responsible for exacerbating pathology.

### Therapeutic intervention with IL-2 Ab Cx activates Teffs in the draining lymph node

**Figure 3:**
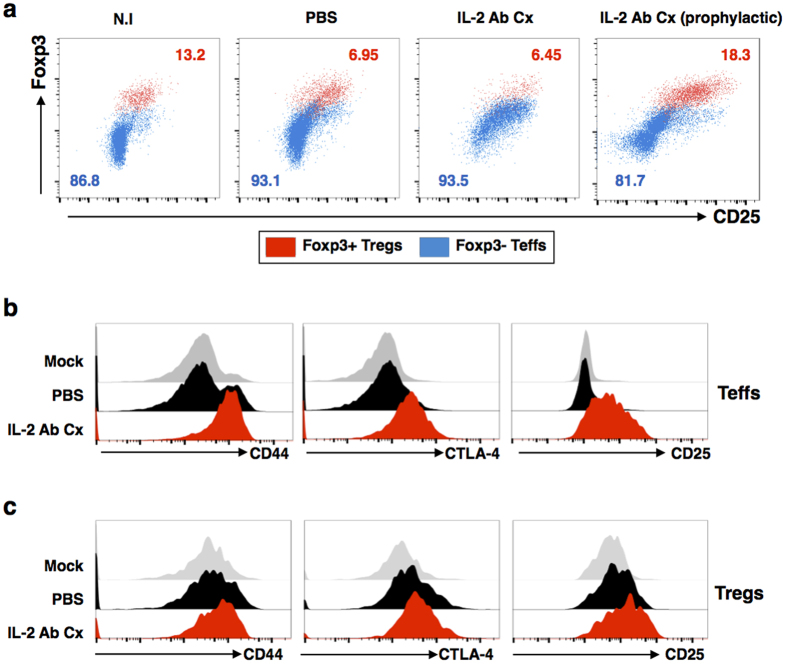
Therapeutic treatment with IL-2 Ab Cx results in activation of Teffs. IL-2 Ab Cx or PBS was administered to CHIKV-infected mice on 3, 4 and 5 dpi. The popliteal lymph node (pLN) was isolated from these mice on 6 dpi. N.I mice were added as a control. Prophylactic treatment of IL-2 Ab Cx was included. **(a)** Representative scatterplots show Foxp3 and CD25 gated on CD4^+^ T cells. Foxp3^+^ Tregs and Foxp3^−^ Teffs are represented in red and blue respectively in the scatterplots. Numbers in scatterplots indicate percentage of Foxp3^+^ Tregs (in red) and Foxp3^−^ Teffs (in blue) in total CD4^+^ T cells. Histogram profiles of activation markers CD44 (left), CTLA-4 (middle) and CD25 (right) on **(b)** Foxp3^−^ Teffs and **(c)** Foxp3^+^ Tregs were also determined. The experiment was performed 3 times independently.

Draining pLN cells were isolated from virus-infected mice on 6 dpi (Fig. 3). While the numbers of Tregs and Teffs in IL-2 Ab Cx-treated mice were not significantly different from the PBS-treated mice (Fig. S4a and e), Teffs were markedly activated after IL-2 Ab Cx treatment (Fig. 3a and Fig. S4b–d). Compared to PBS-treated virus-infected mice (PBS), upregulation of CD25 was observed in the entire Teffs population of IL-2 Ab Cx-treated animals (Fig. 3b and Fig. S4d). Profiling of the Teffs further revealed a marked increase of the activation markers CD44 and CTLA-4 with IL-2 Ab Cx treatment (Fig. 3b and Fig. S4b and c), while the Tregs in pLNs showed only a moderate increase in these activation markers (Fig. 3c and Fig. S4f and g). The latter was a surprising and intriguing observation as splenic Tregs showed very robust proliferation in response to the therapeutic IL-2 Ab Cx treatment (Fig. S1). Expansion of Tregs was accompanied by strong upregulation of activation markers (Fig. S5a–c), similar to what was observed in the prophylactic intervention^22^. Moreover, in contrast to pLN Teffs, splenic Teffs remained completely unresponsive to the IL-2 Ab Cx treatment (Fig. S5). Thus, IL-2 Ab Cx has different effects on the T cell populations in the two compartments during an active virus infection.

### pLN Teffs from virus-infected animals are responsive to IL-2

**Figure 4:**
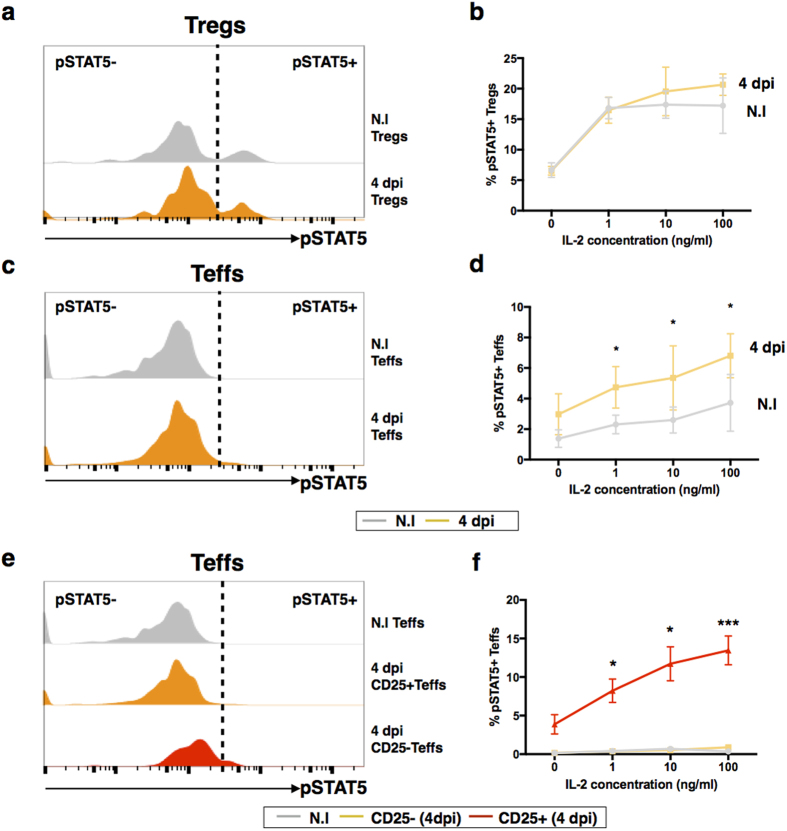
pLN Teffs from virus-infected animals have a hyper response to IL-2. pLN was isolated from CHIKV-infected mice on 4 dpi. N.I. mice were included as a negative control. Stimulation was done using increasing concentration of IL-2. Cells were stained for phospho-STAT5 (pSTAT5) and analyzed with flow cytometry. **(a)** Histogram shows pSTAT5 signaling of Tregs in response to 10 ng/ml IL-2. Dotted line divides the population into pSTAT5^+^ and pSTAT5^−^. **(b)** Line graph show percentage of pSTAT5^+^ Tregs with increasing concentrations of IL-2. **(c)** Histogram shows pSTAT5 signaling of Teffs in response to 10 ng/ml IL-2. Dotted line divides the population into pSTAT5^+^ and pSTAT5^−^. **(d)** Line graph shows percentage of pSTAT5^+^ Teffs with increasing concentrations of IL-2. Dotted line divides the population into pSTAT5^+^ and pSTAT5^−^. All data are presented as mean ± SEM and are derived from 3 independent experiments. Statistical analyzes were performed using unpaired two-tailed Mann-Whitney. (*p = *0.0159* 1 ng/ml IL-2 Teffs, *p = *0.0317* 10 ng/ml IL-2 Teffs, *p = *0.0159* 100 ng/ml IL-2 Teffs. **(e)** Histogram shows pSTAT5 signaling of CD25^+^ and CD25^−^ Teffs from CHIKV-infected mice at 4 dpi in response to 10 ng/ml IL-2. **(f)** Line graph shows percentage of pSTAT5^+^ CD25^+^ and CD25^−^ Teffs from CHIKV-infected mice at 4 dpi with increasing concentrations of IL-2. Dotted line divides the population into pSTAT5^+^ and pSTAT5^−^. All data are presented as mean ± SEM and are derived from 3 independent experiments. Statistical analyses were performed using Kruskal-Wallis followed by Dunn’s multiple comparison test comparing to N.I. mice. (*p = 0.0236 for 1 ng/ml IL-2 CD25^+^ Teffs, *p = 0.0236 for 10 ng/ml IL-2 CD25^+^ Teffs, ***p = 0.0008 for 100 ng/ml IL-2 CD25^+^ Teffs).

In contrast to Teffs, Tregs constitutively express CD25 (IL-2Rα) on their surface^31^. Presence of IL-2Rα is known to confer high IL-2 affinity to the heterotrimeric IL-2R complex^32^,^33^. The signal induced through interactions of IL-2 and IL-2R is mediated through Jak-STAT5 pathway^34^. Phosphorylation of STAT5 (pSTAT5) is one of the earliest events in IL-2R signaling^35^,^36^ and can be measured *in vitr*o by flow cytometry. In healthy non-infected (N.I) mice, only splenic Tregs show phosphorylation of STAT5 at low IL-2 concentrations. In Teffs, pSTAT5 is detectable at very high IL-2 concentration in only a small fraction of the population (Fig. S6). The fraction of pSTAT5^+^ cells was substantially higher Tregs compared to Teffs (Fig. S6). However, no significant difference in pSTAT5 signaling was observed in the Tregs between N.I and virus-infected mice (Fig. 4a,b). On the other hand, a higher level of pSTAT5 signaling was observed in Teffs from virus-infected mice as compared to the N.I mice (Fig. 4c,d). On further analysis, it is revealed that majority of the Teffs from CHIKV-infected mice that responded to IL-2 are CD25^+^ (Fig. 4e,f). Thus Teffs from virus-infected mice are more sensitive to IL-2 signaling, and hence are also more likely to respond to the IL-2 Ab Cx treatment.

### A large fraction of human Teffs is sensitive to IL-2

**Figure 5:**
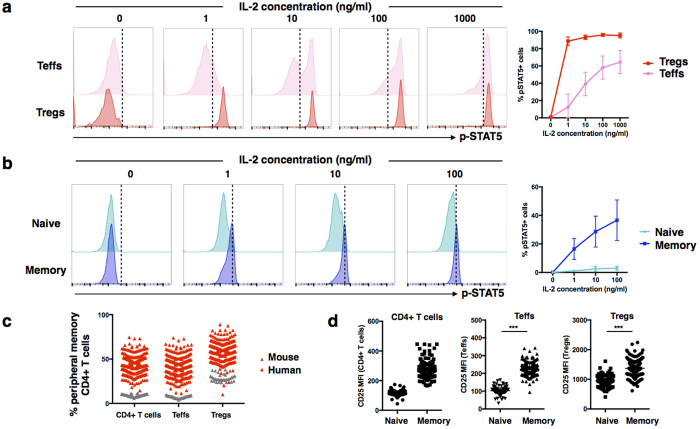
Human Teffs respond efficiently to low concentrations of IL-2. Peripheral blood mononuclear cells (PBMCs) were isolated from 7 healthy donors and stimulated with increasing concentrations of human IL-2 (1 to 1000 ng/ml) stimulation. pSTAT5 signaling in CD4^+^ T cells were measured. **(a)** Representative histograms of pSTAT5 signaling in Teffs (top) and Tregs (bottom) in response to increasing concentration of IL-2. Line graph shows average percentage of pSTAT5^+^ Tregs and Teffs with respect to increasing concentration of IL-2 stimulation. **(b)** Representative histograms of pSTAT5 signaling in naive (CD45RA^+^) (top) and memory (CD45RA^−^) (bottom) CD4^+^ T cells in response to increasing concentration of IL-2. Line graph shows average percentage of pSTAT5^+^ naive and memory CD4^+^ T cells with respect to increasing concentration of IL-2 stimulation (n = 4). Data was presented as mean ± SD. **(c)** Blood was collected from 261 healthy donors and assessed for the percentage of memory population (CD45RA^−^) in peripheral CD4^+^ T cells, CD4^+^ Teffs and CD4^+^ Tregs using flow cytometry. The percentage of peripheral memory CD4^+^ T cells population (CD44^+^) in mice was also assessed in comparison. **(d)** CD25 expression on naïve (CD45RA^+^) and memory (CD45RA^−^) population from **(c)** was also measured.

In naïve mice, typically all of the IL-2 responsive cells are Tregs (Fig. S4a). To determine if the same applies in humans, a series of IL-2 stimulations was done on human peripheral mononuclear cells (PBMCs) obtained from healthy donors. While human Tregs are extremely sensitive to IL-2, (Fig. 5a), a large fraction of the Teffs was also able to respond well to IL-2. Further characterization revealed that the majority of Teffs that responded are of the memory (CD45RA^−^) phenotype (Fig. 5b). In contrast, the proportion of naïve (CD45RA^+^) Teffs that responded to even high levels of IL-2 are negligible (Fig. 5b). This observation could explain the low response in the N.I. mice to IL-2 (Fig. 4a).

A major difference between humans and laboratory mice is the lifespan as well as the level of exposures to environmental antigens^37^,^38^. Using CD44 as a marker for murine memory T cells^39^,^40^, less than 10% of circulating CD44^+^ CD4^+^ T cells was detected in N.I mice (Fig. 5c). In contrast, this fraction is substantially higher in healthy human adults (Fig. 5c). Moreover, this proportion varied widely within the population, with some individuals having as high as 80% of circulating memory T cells (Fig. 5c). In addition, CD25 expression is also significantly higher in these memory T cells (Fig. 5d).

As IL-2 preferentially stimulates the memory population (Fig. 5b), this suggest that administration of IL-2 Ab Cx is can trigger strong pro-inflammatory responses even in healthy individuals.

## Discussion

We previously demonstrated that prophylactic treatment with IL-2 Ab Cx prevents the development of arthralgia after CHIKV infection^21^. It prompted us to assess this approach under therapeutic conditions. Surprisingly, this regimen not only failed to prevent inflammation, it even led to an exacerbated inflamed joint pathology associated with massive infiltration of highly activated pathogenic CD4^+^CD25^+^ Teffs.

The IL-2 Ab Cx has been used in a number other mouse models of infection with different outcomes^41^,^42^,^43^,^44^,^45^. However, the conversion of the anti-inflammatory into a pro-inflammatory effect during therapeutic applications has yet to be reported. In the malaria model, administration of the antibody complex right after acute malaria infection at 0 to 2 days resulted in increased Treg numbers^42^. However, in the EAE model, administration of the antibody complex 7 days after MOG induction (3 days after disease onset) resulted in the loss of protection^43^. In our study, the complex was administered just before the peak of disease, when the clinical symptoms have already manifested in the form of joint inflammation. The CD4^+^ Teffs in the draining pLNs have already expressed high levels of CD25 and this rendered them receptive to the IL-2 Ab Cx, which resulted in expansion and activation of pathogen-specific Teffs. Notably, the direction of outcome of the IL-2 Ab Cx treatment strongly depends in the compartment. Although a similar selective expansion of Tregs was observed in the spleen and blood^21^, an accumulation of highly activated Teffs was observed in the draining pLNs.

The heightened sensitivity of these cells to IL-2 was associated with upregulation of CD25, the IL-2Rα chain that confers high affinity to IL-2R^32^,^33^. The X-ray crystal structure of IL-2 Ab Cx revealed steric hindrance of JES6-1 on IL-2Rβ (CD122) and IL-2Rγ (CD132) chains^46^. The affinity of IL-2 for IL-2βγ is lower than JES6-1, but increases significantly in the presence of IL-2Rα to displace JES6-1 from IL-2^46^. This in turn results in the ‘hand over’ of IL-2 from JES6-1 to the high affinity IL-2 Rαβγ present on activated Teffs. In addition, IL-2 signaling increases expression of IL-2Rα, thereby providing a continuous transcriptional feedback loop leading to the enhanced Teffs activation^46^,^47^.

In contrast to laboratory mice, healthy humans already have a large fraction of CD25-expressing memory cells that are poised to react. IL-2 has been used to treat cancer in patients since the 1980 s^48^. While it was successful in halting the progression of metastatic melanoma, severe side effects like vascular leakage syndrome (VLS) were reported^49^. On the other hand, treatment with low dose IL-2 has been met with some success, whereby it could reverse Graft-versus host disease (GvHD) in patients^50^. Similarly, treatment with low dose IL-2 could improve vasculitis in patients infected with Hepatitis C virus^51^,^52^. These improvements were associated with an increased in Tregs as a result of the low dose IL-2 treatment^50^,^51^.

While treatment with IL-2 has been relatively well documented, the use of IL-2 Ab Cx has never been reported in clinical trials. Although anti-CD3^53^,^54^ or anti-CD4 antibody treatments^55^,^56^ have been used successfully for Tregs-directed immunotherapy in humans, agonistic immune-modulatory reagents carry intrinsic risks. In pre-clinical trials the use of agonistic anti-CD28 (TGN1412) to expand Tregs was highly successful in the treatment of experimental autoimmune encephalomyelitis and adjuvant arthritis in rodent models^57^,^58^,^59^,^60^. However, all of the healthy volunteers involved in the phase I clinical trial suffered from a severe systemic inflammatory response syndrome (SIRS) within 90 min of intravenous (IV) administration^61^. The phenomenon was likely due to the large proportion of effector-memory T cells (T_EM_ cells) present in human blood that responded immediately to anti-CD28 stimulation by releasing pro-inflammatory cytokines such as IFNγ, TNFα and IL-2^37^,^57^.

The anti-CD28 clinical trial highlighted fundamental differences between humans and laboratory rodents that should be taken into account when interpreting pre-clinical data. Unlike murine Teffs, a large proportion of human Teffs respond to low concentrations of IL-2 due to the presence of memory cells. Life expectancy of humans average around 70 yrs, allowing large accumulation of memory cells due to multiple exposures to environmental stimulus and pathogens^62^. These CD25-expressing memory cells are poised to respond immediately to strong T cell stimulus^63^. Thus, using IL-2 Ab Cx in this environment is likely to cause adverse effects by driving the activation and expansion of Teffs. Nevertheless, due to the crucial role of Tregs in controlling inflammatory pathologies^21^,^23^,^43^,^64^, it is worthwhile to continue exploring other methods that allow for a selective expansion of Tregs as treatment for inflammatory diseases^65^.

## Methods

### Virus stocks

CHIKV isolate (SGP011) used for all infections was isolated from the 2008 outbreak in Singapore at the National University Hospital. The virus was propagated in C6/36 and titer of SGP011 was determined using standard plaque assays with Vero-E6 cells^66^,^67^.

### Animal studies

Three-week-old WT or *CD4* KO C57/BL6J female mice were inoculated subcutaneously in the ventral side of the right hind footpad with 10^6^ pfu CHIKV diluted in 30 μl PBS. Following CHIKV infection, PBS, 1.5 μg murine IL-2 (Peprotech), 50 μg of rat anti-mouse IL-2 (JES6-1) monoclonal antibody (eBioscience) or 1.5 μg murine IL-2 in complex with 50 μg anti-mouse IL-2 mAb were injected i.p daily on 3, 4 and 5 dpi. IL-2 Ab Cx pre-treatment was carried out as described^21^. Joint footpad swelling was determined daily by the measurement of the height and the breadth of the footpad using a caliper and quantified as (height x breadth). The degree of inflammation was expressed as relative increase compared to pre-infection (d 0) with the following formula: [(x –d 0)/d 0] where x is footpad size measurements for respective dpi^4^,^25^,^27^,^66^.

### Viremia measurement

Ten microliters of blood was collected daily from the tail vein and viral RNA were extracted using QIAamp^**®**^ Viral RNA Mini Kit (QIAGEN) following manufacturer’s protocol. Quantification of viral load was done with QuantiTect^®^ Probe RT-PCR (QIAGEN) using 7900HT Fast Real-Time PCR System machine (Applied Biosciences) with thermal cycling conditions as described previously^4^,^25^,^27^,^66^,^68^. The limit of detection is 10 RNA copies/μl.

### Blood leukocyte immunophenotyping

Fifteen microliters of blood were collected from the tail vein. Briefly, blood was lysed using 1X Mouse Lyse Buffer (R&D Systems) and washed twice with PBS. Cells were stained with rat anti-CD4 (clone RM4–5, BD Biosciences), rat anti-CD25 (PC61.5, eBioscience), rat anti-CD44 (IM7, eBioscience) antibodies. Intracellular staining of Foxp3 was done using Foxp3 staining buffer set (eBioscience) and rat anti-Foxp3 (FJK-16s, eBioscience) following manufacturer’s instructions. Data was acquired using BD LSRFortessa™ cell analyzer. Dead cells and duplets were excluded in all analyzes using forward and side scatter gating. Results were analyzed with FlowJo version X software (Tree Star, Inc).

### Tissue leukocyte Immunophenotyping

Spleen, popliteal lymph node (pLN) and joint footpad of CHIKV-infected mice were harvested at 6 dpi and 7 dpi. Isolation of splenocytes, pLN and joint footpad cells were processed as described^4^,^21^,^26^. Isolated cells from spleen, joint footpad and pLN were first stained with Live/dead Fixable Aqua Dead Cell Stain Kit (1:400) (Life Technologies) for 20 min. Cell were then washed and resuspended in staining buffer (2% fetal bovine serum in PBS). Staining was performed using rat anti-CD45 (30-F11, BD Pharmingen), rat anti-CD4 (RM4–5, BD Pharmingen), rat anti-CD3 (17A2, Biolegend), rat anti-CD25 (PC61.5, eBioscience), hamster anti-CTLA-4 (UC10-4B9, eBioscience) and rat anti-CD44 (IM7, eBioscience) antibodies for 15 min. Stained cells were then washed with PBS and fixed using IC Fixation Buffer (eBioscience). Intracellular staining of rat anti-Foxp3 (FJK-16s, eBioscience) was done according to manufacturer’s instructions. Data acquisition and analyzes were done as described above.

### Detection of phosphorylated STAT5 (phospho-STAT5) signalling

Murine splenocytes or pLN were isolated as described above. Isolated leukocytes were resuspended in complete RPMI medium (10% fetal bovine serum (FBS) in RPMI medium) containing 0, 1, 10 or 100 ng/ml murine IL-2 (Peprotech). Stimulation was performed at 37 °C for 30 mins. Stimulation was terminated by the addition of 1X BD Phosflow^TM^ Lyse/Fix Buffer (BD Bioscience) at 37 °C for 10 mins. Permeabilization was performed using Perm Buffer III (BD Bioscience) on ice for 30 mins. Cells were then washed 3 times with staining buffer to completely remove traces of Perm Buffer III before proceeding to stain with rat anti-CD4 (RM4–5, BD Bioscience), rat anti-CD25 (7D4, eBioscience), rat anti-Foxp3 (FJK-16s, eBioscience) and anti-pSTAT5 (pY694) (BD Bioscience) antibodies for 1 hr. Human peripheral blood mononuclear cells (PBMCs) were obtained from blood of healthy donors using Ficoll-Plaque (GE Healthcare)^67^. Stimulation of PBMCs was done as above except that human IL-2 (Peprotech) was used and stimulation was done at 37 °C for 15 mins instead. Staining was done using mouse anti-human CD3 (UCHT1, BD Bioscience), mouse anti-human CD4 (SK3, BD Bioscience), mouse anti-human CD45RA (HI100, eBioscience), rat anti-human Foxp3 (PCH101, BD Bioscience) and mouse anti-pSTAT5 (pY694, BD Bioscience) antibodies for 1 hr.

### Peripheral human memory CD4^+^ T cells determination

Two hundred and sixty-one healthy donors were recruited in a cohort study and PBMCs were isolated as described^69^. The percentage of CD4^+^CD45RA^−^ population was gated as described^69^ and determined by 100 – x %CD4^+^CD45RA^+^ T cells as adult peripheral T cells are either CD45RA^+^ or CD45RO^+^ 70. The MFI of CD25 on naïve (CD45RA^+^) and memory (CD45RA^−^) CD4^+^ T cells was also determined as previously described^69^.

### Statistical analysis

All statistical analyzes were performed using Graphpad Prism 6. All analyzes between PBS, IL-2, JES6-1 and IL-2 Ab Cx (prophylatic) were done using Kruskal-Wallis followed by Dunn’s post-test comparing all groups to PBS. Comparison between particle analysis of PBS and IL-2 Ab Cx was done using Mann Whitney *U* test. A *p* value of less than 0.05 is considered to be statistically significant.

### Study approval

All mice were handled in strict accordance with good animal practice as defined by the National Advisory Committee for Laboratory Animal Research (NACLAR) guidelines licensed by the Agri-Food and Veterinary Authority of Singapore (AVA). Mice were housed in the ABSL2 facility at the Biological Resource Center (BRC) at Biopolis, Singapore. All studies were reviewed and approved by the A*STAR BRC Institutional Animal Care and Use Committee (IACUC) under IACUC No. 151038. All sections of this study adhere to and were performed in accordance to IACUC protocol No. 151038. All studies performed on human samples have been approved by the IRB of the National University of Singapore (IRB ref. NUS IRB 10–445) and complied with the Helsinki Declaration. Written informed consent was obtained from all donors prior to sample collection.

## Additional Information

**How to cite this article**: Lee, W. W. L. *et al*. Virus infection drives IL-2 antibody complexes into pro-inflammatory agonists in mice. *Sci. Rep*. **6**, 37603; doi: 10.1038/srep37603 (2016).

**Publisher's note:** Springer Nature remains neutral with regard to jurisdictional claims in published maps and institutional affiliations.

## Supplementary Material

Supplementary Information
