# Supplementary material for: Virus infection drives IL-2 antibody complexes into pro-inflammatory agonists in mice
Source: Sci Rep. 2016 Nov 25;6:37603. doi: 10.1038/srep37603 (PMC5122839; doi:10.1038/srep37603)
Supplement: Supplementary Information [file srep37603-s1.pdf]

Scientific Reports

Supplemental Materials

Virus infection drives IL-2 antibody complexes into pro-inflammatory agonists in mice

Wendy W.L. Lee[1,2], Teck-Hui Teo[1,2], Fok-Moon Lum[1,3], Anand K Andiappan[1], Siti Naqiah Amrun[1], Laurent Rénia[1,4], Olaf Rötzschke[1,4,*,+], and Lisa F.P. Ng[1,3,*,+]

[1] Singapore Immunology Network, Agency for Science, Technology and Research, Singapore (A*STAR), Singapore

[2] NUS Graduate School for Integrative Sciences and Engineering, National University of Singapore, Singapore

[3] Department of Biochemistry, Yong Loo Lin School of Medicine, National University of Singapore, Singapore

[4] Department of Microbiology, Yong Loo Lin School of Medicine, National University of Singapore

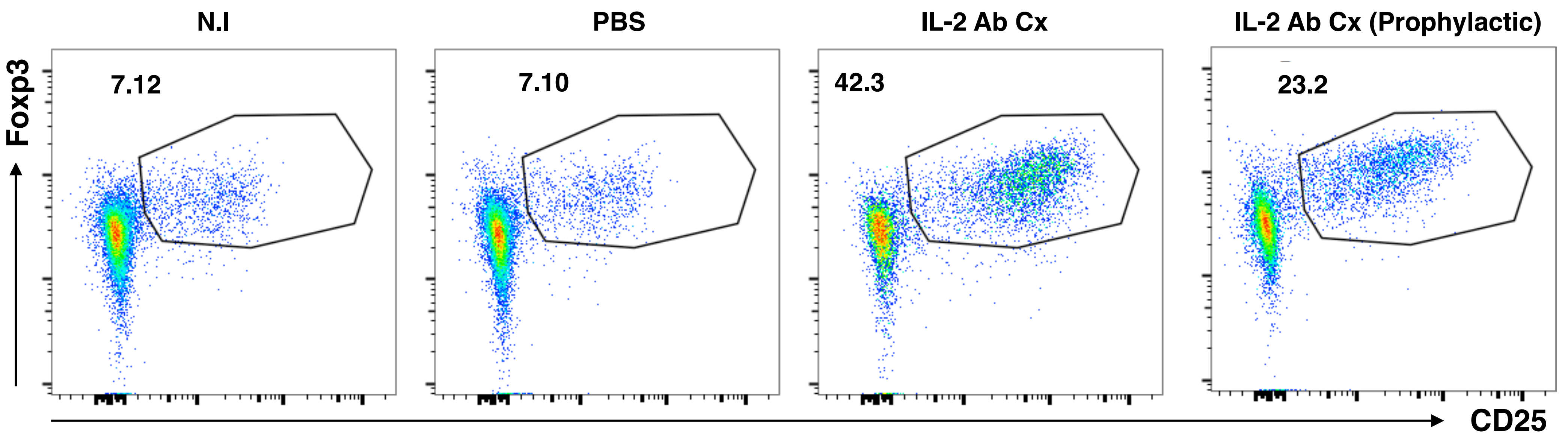

Supplementary Fig. 1: Treatment with IL-2 Ab Cx after infection results in expansion of splenic Tregs. IL-2 Ab Cx or PBS was administered to CHIKV-infected mice on 3, 4 and 5 dpi (n=5 per group). The spleen was isolated from these mice on 6 dpi. N.I was added as a control. Prophylaxis treatment of IL-2 Ab Cx was also included. Figure shows scatterplot of Foxp3 versus CD25 expression. Population in the boxed region denotes Foxp3+CD25+ Tregs and number in the box indicates the percentage Tregs of total CD4+. Data presented are representative of 3 independent experiments

Supplementary Fig. 1 Lee *et al.*, 2016

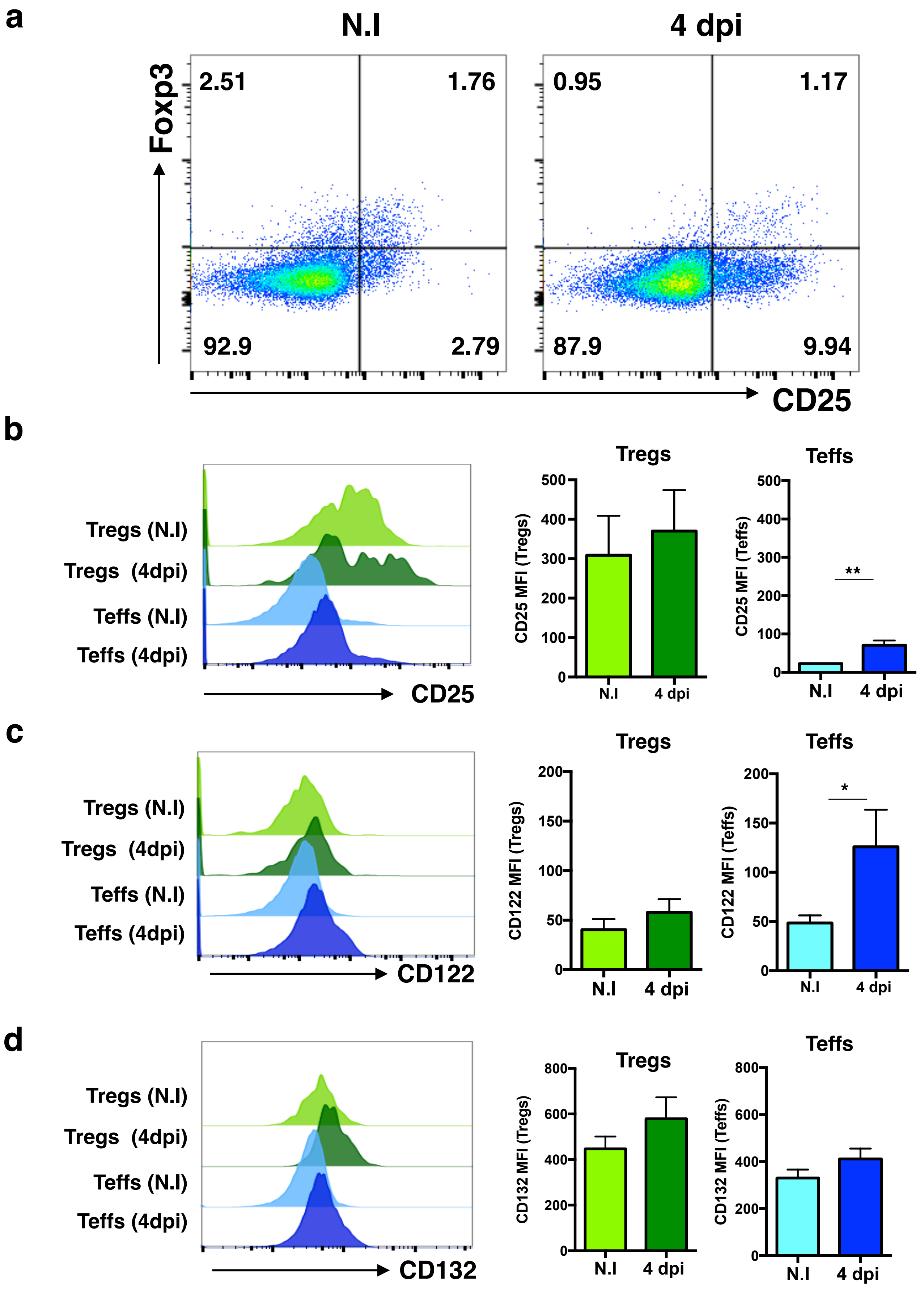

Supplementary Fig. 2: CHIKV infection increases expression of CD25 and CD122 on Teffs. **(a)** Scatterplots show the expression of Foxp3 and CD25 on CD4+ T cells isolated from pLN of CHIKV-infected mice. N.I mice were included as negative control. Numbers in the boxes indicate the percentage of cells in the respective quadrants. The presented data is representative of 3 independent experiments. Representative hisotgram and bar charts of **(b)** CD25, **(c)** CD122 and **(d)** CD132 expression on Tregs and Teffs were measured. Statistical analysis was performed using one tail unpaired t test (n=3 per group). ***p<0.0013* Teffs CD25, **p<0.0126* Teffs CD122)

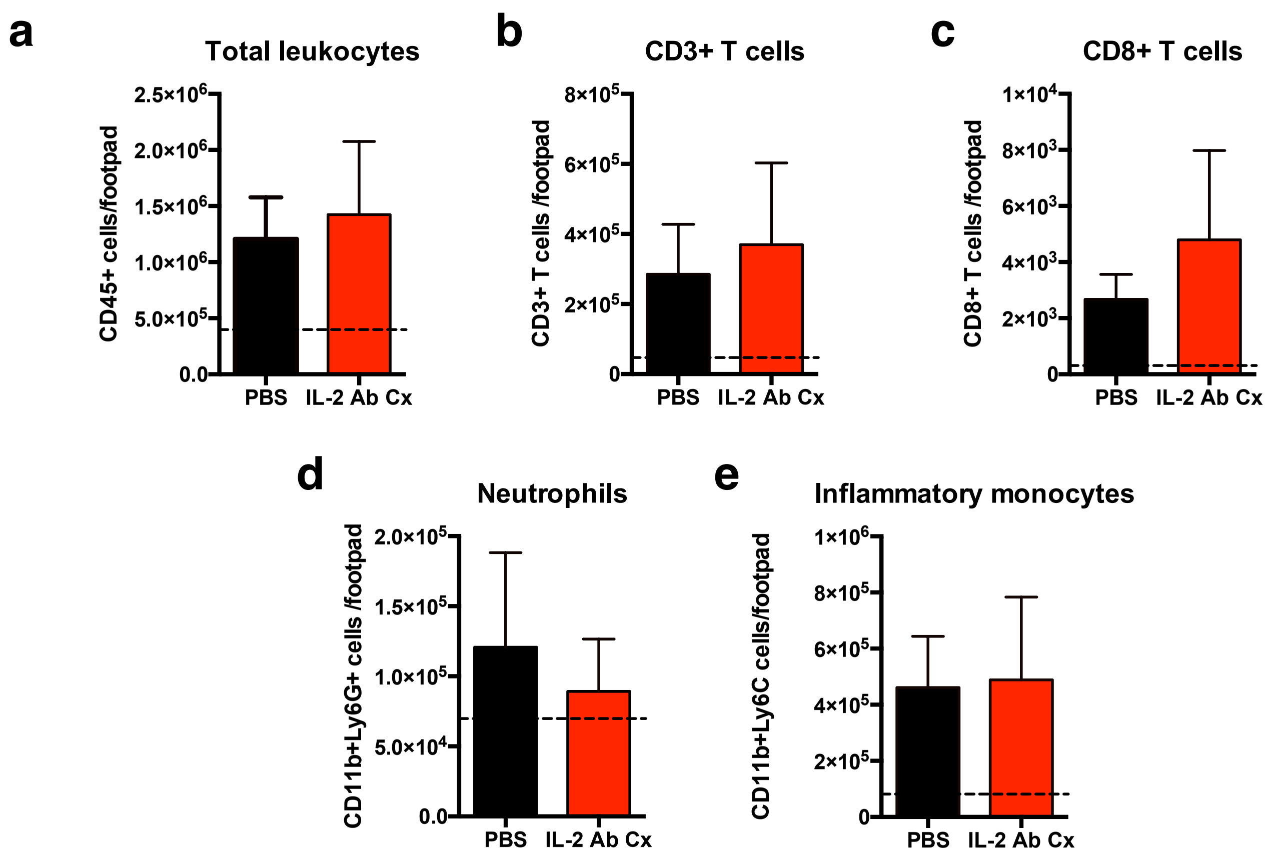

Supplementary Fig. 3: WT mice (n=5 per group) were infected s.c with 10^6 PFU CHIKV and administered with either PBS or IL-2 Ab Cx. Joint footpad cells from treated animals were isolated on 7 dpi and analyzed by flow cytometry. Dead cells were excluded using Live/dead staining. Bar chart showing average number of **(a)** total leukocytes, **(b)** CD3+ T cells, **(c)** CD8+ T cells, **(d)** neutrophils and **(e)** inflammatory monocytes per infected footpad. All data are presented as mean ± SD and from 3 independent experiments. Statistical analysis was performed using two-tailed Mann Whitney *U* test comparing between PBS and IL-2 Ab Cx.

Supplementary Fig. 3 Lee *et al.*, 2016

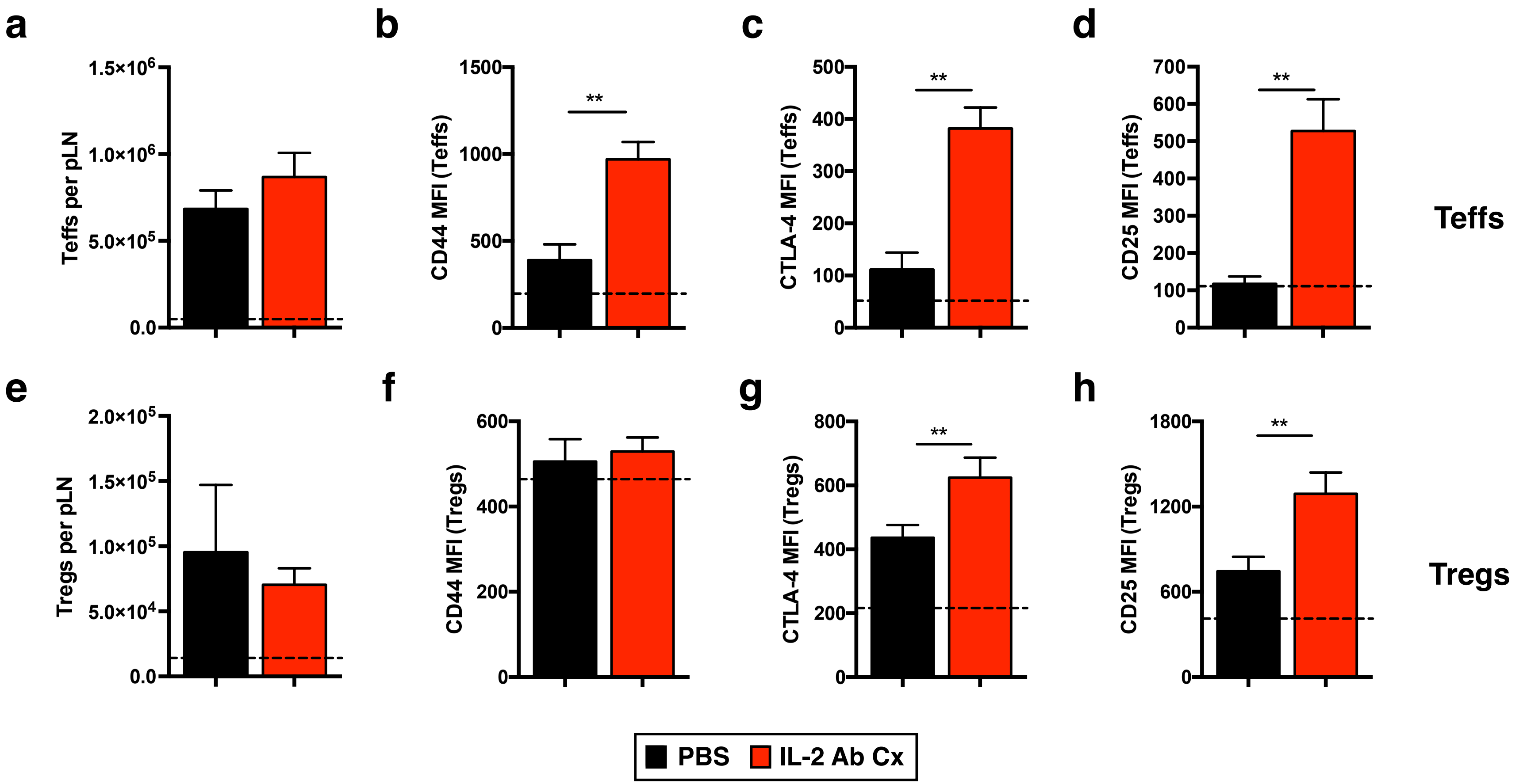

Supplementary Fig. 4: IL-2 Ab Cx or PBS was administered to CHIKV-infected mice on 3, 4 and 5 dpi. The pLN was isolated from these mice on 6 dpi. Bar charts show **(a)** average number of Teffs and the MFI of **(b)** CD44, **(c)** CTLA-4 and **(d)** CD25 on these cells. Bar chart show **(e)** average number of Tregs and the MFI of **(f)** CD44, **(g)** CTLA-4 and **(h)** CD25 on these cells. Dotted line indicates average of N.I. All data are presented as mean ± SD and representative of 3 independent experiments. Statistical analysis was performed using two-tailed Mann Whitney *U* test comparing between PBS and IL-2 Ab Cx. (**p=0.0079 CD44 Teffs, **p=0.0079 CTLA-4 Teffs, **p=0.0079 CD25 Teffs, **p=0.0079 CTLA-4 Tregs **p=0.0079 CD25 Tregs)

Supplementary Fig. 4 Lee *et al.*, 2016

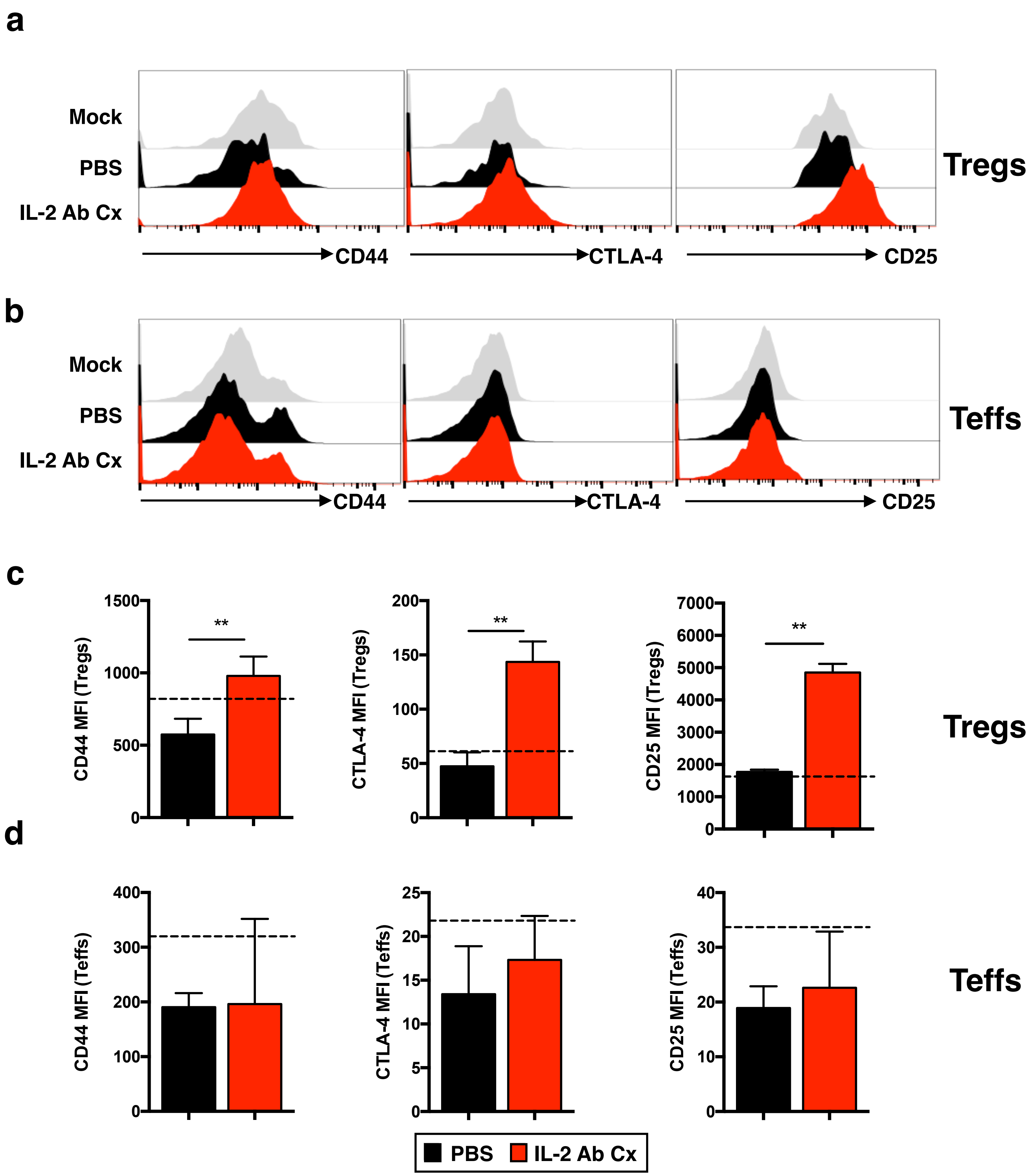

Supplementary Fig. 5: IL-2 Ab Cx or PBS was administered to CHIKV-infected mice on 3, 4 and 5 dpi. The spleen was isolated from these mice on 6 dpi. Representative histograms show CD44, CTLA-4 and CD25 expression on **(a)** Tregs and **(b)** Teffs respectively. **(b)** Bar charts indicate the average MFI of CD44, CTLA-4 and CD25 on **(c)** Tregs and **(d)** Teffs respectively. Dotted lines indicate average of N.I. All data are presented as mean ± SD and representative of 3 independent experiments. (**p=0.0079 CD44 Tregs **p=0.0079 CTLA-4 Tregs, **p=0.0079 CD25 Tregs)

Supplementary Fig. 5 Lee *et al.*, 2016

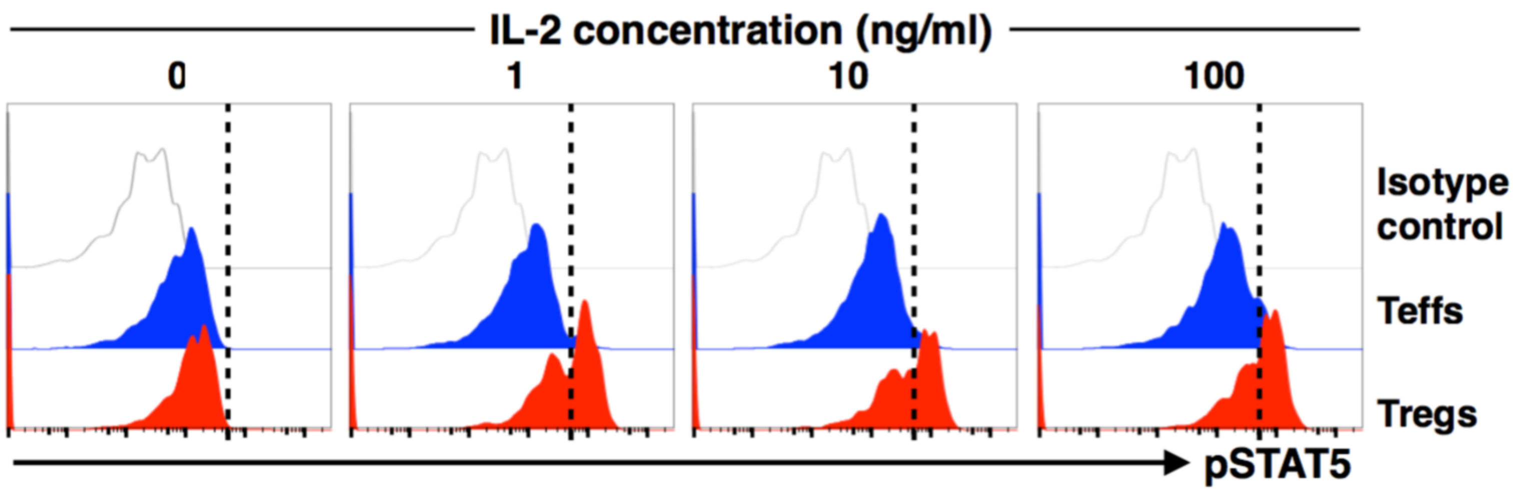

Supplementary Fig. 6: Splenocytes from N.I mice were stimulated with increasing concentration of murine IL-2 and pSTAT5 signaling was compared between Teffs and Tregs in histogram plots. Dotted lines were drawn to indicate pSTAT5+ population.

Supplementary Fig. 6 Lee *et al.*, 2016
